# Laser Powder Bed Fusion of Polymers: Quantitative Research Direction Indices

**DOI:** 10.3390/ma14051169

**Published:** 2021-03-02

**Authors:** Ihsan Murat Kusoglu, Carlos Doñate-Buendía, Stephan Barcikowski, Bilal Gökce

**Affiliations:** 1 Technical Chemistry I, Center for Nanointegration Duisburg-Essen (CENIDE), University of Duisburg, 45141 Essen, Germany; ihsan.kusoglu@uni-due.de (I.M.K.); carlos.donate-buendia@uni-due.de (C.D.-B.); bilal.goekce@uni-due.de (B.G.); 2 Materials Science and Additive Manufacturing, School of Mechanical Engineering and Safety Engineering, University of Wuppertal, 42119 Wuppertal, Germany

**Keywords:** additive manufacturing, 3D-printing, selective laser sintering, SLS, PA12, PEEK, nano, additives, bibliometry

## Abstract

Research on Laser Powder Bed Fusion (L-PBF) of polymer powder feedstocks has raised over the last decade due to the increased utilization of the fabricated parts in aerospace, automotive, electronics, and healthcare applications. A total of 600 Science Citation Indexed articles were published on the topic of L-PBF of polymer powder feedstocks in the last decade, being cited more than 10,000 times leading to an h-index of 46. This study statistically evaluates the 100 most cited articles to extract reported material, process, and as-built part properties to analyze the research trends. PA12, PEEK, and TPU are the most employed polymer powder feedstocks, while size, flowability, and thermal behavior are the standardly reported material properties. Likewise, process properties such as laser power, scanning speed, hatch spacing, powder layer thickness, volumetric energy density, and areal energy density are extracted and evaluated. In addition, material and process properties of the as-built parts such as tensile test, flexural test, and volumetric porosity contents are analyzed. The incorporation of additives is found to be an effective route to enhance mechanical and functional properties. Carbon-based additives are typically employed in applications where mechanical properties are essential. Carbon fibers, Ca-phosphates, and SiO_2_ are the most reported additives in the evaluated SCI-expanded articles for L-PBF of polymer powder feedstocks. A comprehensive data matrix is extracted from the evaluated SCI-index publications, and a principal component analysis (PCA) is performed to explore correlations between reported material, process, and as-built parts.

## 1. Introduction

Recent progress, as well as commercialization trends, in the Additive Manufacturing (AM) industry can be extracted from survey-based commercial reports [1] and peer-reviewed publication-based bibliometric analysis [2]. The high impact of AM in a wide variety of sectors such as aerospace [3], automotive [4,5], electronics [6], and healthcare [7] has attracted the attention of many research and development activities that are further contributing to the progress of AM techniques and applications of 3D-printed polymer parts. The current research and industrial trends aim to develop new powder feedstock materials, improve the already available powders’ processability, enhance the properties, or provide new functionalities to the as-built parts [3,4,5,8,9]. The results arising from those research topics are reported in several databases. Consequently, a systematic evaluation of the data represents a useful approach to elucidate and provide an initial framework for future trends in the field of AM. Here, the analysis of indexed publication databases presents an alternative to survey-based trend analysis. SCI-expanded publications are trustable report sources since these publications are peer-reviewed, quality-assayed, and ensure unbiased information. In this sense, a first analysis of the SCI-Expanded articles on AM produced within the last decade shows the rapid growth of the number of publications, proving a particular interest in one of the sub-class techniques, i.e., laser powder bed fusion (L-PBF). As stated in the EN ISO/ASTM 52900:2018 [10], powder bed fusion of polymer powder feedstock using a laser beam (PBF-LB/P) is defined as an additive manufacturing process that leads to the fabrication of polymer parts by laser sintering.

This study aims to provide a comprehensive evaluation of the reported properties of the material, the process, and the as-built parts from the 100 most cited SCI-expanded articles on L-PBF of polymer powder feedstocks published over the last decade. This way, a precise evaluation of the research trends in the field can be provided, as well as a framework for the optimization of the material, process parameters, and additives selection in L-PBF for the manufacturing of the desired as-built parts.

## 2. Approach and Bibliometric Method

**Figure 1:**
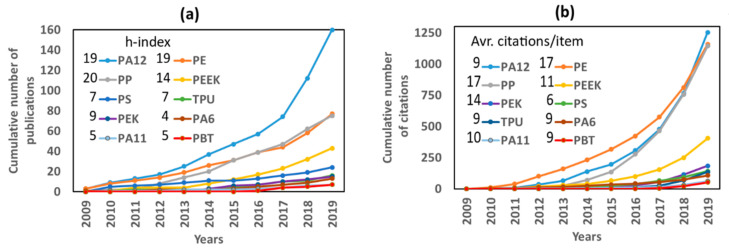
Bibliometrics on L-PBF of PA12, PA11, PA6, PP, PEEK, TPU, PEK, PS, PE, and PBT powder feedstocks processed by L-PBF over the last decade: (**a**) cumulative number of publications and corresponding h-index; (**b**) the cumulative number of citations and average citations per item.

The number of SCI-Expanded publications on L-PBF of several polymer powder feedstocks with their reported citations and h-indices was searched using the Web of Science (WoS) Core Collection database. The evaluation of the publications was carried out over the period ranging from 2009 to 2019. The details of the publication set and their results are provided as supplementary information in Table S1. Almost 2500 publications were reported in the field of AM of polymers, which have been cited 42,500 times with an h-index of 84. As can be observed in Table S1, “powder bed fusion” (PBF), “selective laser sintering” (SLS), “selective laser melting” (SLM), “laser sintering” (LS), and “laser beam melting” (LBM) were set as search strings to find out the status of the SCI-expanded publications on a layer-by-layer laser processing of polymer powder feedstocks. Through the last decade, over 600 SCI-expanded publications reported L-PBF of polymers, and those have been cited more than 10,000 times, which corresponds to an h-index of 46. Likewise, PBF, SLS, SLM, LS, and LBM search strings were combined with several types of polymers, such as polyamide 12 (PA12), polyamide 11 (PA11), polyamide 6 (PA6), polyether ether ketone (PEEK), polyether ketone (PEK), polypropylene (PP), polyethylene (PE), polystyrene (PS), and polybutylene terephthalate (PBT), the results are given in Table S1. Several polymer powder feedstock types are already commercially available in the market, such as polyamides (PA12, PA11), polyether ether ketone (PEEK), thermoplastic polyurethane (TPU), polypropylene (PP), and polystyrene (PS). The polymer selection is based on the desired application of the generated part, as each of them presents different thermophysical or mechanical properties. The cumulative number of publications, citations, h-indices, and average citations per item of these polymer types processed by L-PBF are shown in Figure 1.

SCI-expanded publications on L-PBF of polymer powder feedstocks have been increasing over the last decade. PA12 is the most studied polymer powder feedstock for L-PBF, with 160 publications and an h-index of 19. Interestingly, a search in Google Scholar with the keywords “selective laser sintering” and “polyamide 12” (patents not included) leads to approx. 1500 results. This difference indicates that while there is a significant number of research studies on L-PBF of PA12, many publications in that field are not published in SCI-indexed journals.

Starting with the comparison of the materials employed, the Web of Science search shows that between 2015 and 2019, there was notable growth in the total number of publications for PA12, PE, PP, PEEK, and h-indices of these polymers were almost twice as much as for the other searched polymers (Figure 1a). Even though the number of reported publications on PA12 was a factor 2 higher than for PE and PP, h-indices had the same value. The citations’ total number was almost identical for these polymer powder feedstocks, ranging between 1150 and 1250. Accordingly, reports on L-PBF of PE and PP are the most visible ones with an average citation per item of 17, which was almost double of PA12. PEEK, which is a high-performance semi-crystalline polymer, was the fourth most cited polymer powder feedstock with 405 citations over the last decade.

**Figure 2:**
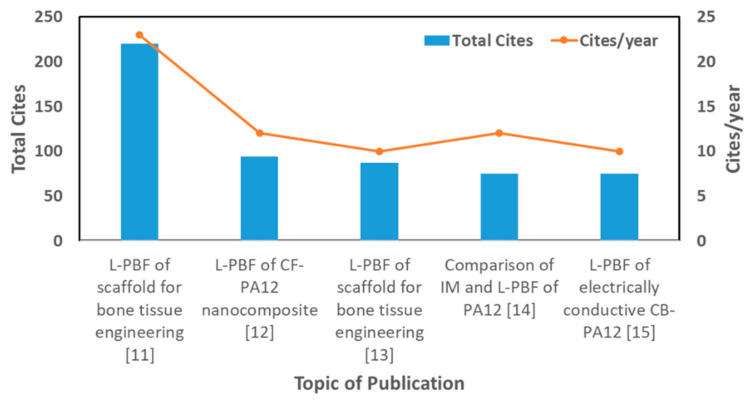
The topic of the five most cited SCI-expanded publications of the statistically evaluated references [11,12,13,14,15,16,17,18,19,20,21,22,23,24,25,26,27,28,29,30,31,32,33,34,35,36,37,38,39,40,41,42,43,44,45,46,47,48,49,50,51,52,53,54,55,56,57,58,59,60,61,62,63,64,65,66,67,68,69,70,71,72,73,74,75,76,77,78,79,80,81,82,83,84,85,86,87,88,89,90,91,92,93,94,95,96,97,98,99,100,101,102,103,104,105,106,107,108,109,110,111] and their average citations per year over the last decade.

Among the 600 publications reported for L-PBF of polymer powder feedstocks, a general analysis in terms of citations, materials employed, and temporal evolution of the number of publications has been provided in Figure 1 and Table S1. A subgroup from the 600 publications is chosen to perform a detailed statistical analysis. The selection criteria employed is to analyze the 100 most cited research articles [11,12,13,14,15,16,17,18,19,20,21,22,23,24,25,26,27,28,29,30,31,32,33,34,35,36,37,38,39,40,41,42,43,44,45,46,47,48,49,50,51,52,53,54,55,56,57,58,59,60,61,62,63,64,65,66,67,68,69,70,71,72,73,74,75,76,77,78,79,80,81,82,83,84,85,86,87,88,89,90,91,92,93,94,95,96,97,98,99,100,101,102,103,104,105,106,107,108,109,110,111] published over the last decade within the polymer L-PBF bibliography. The detailed statistical analysis is based on evaluating the reported properties from each of the selected papers, excluding the review articles from the selection criteria. As a first approach, Figure 2 shows the research topics of the five most cited research articles for L-PBF of polymers in the evaluated references [11,12,13,14,15,16,17,18,19,20,21,22,23,24,25,26,27,28,29,30,31,32,33,34,35,36,37,38,39,40,41,42,43,44,45,46,47,48,49,50,51,52,53,54,55,56,57,58,59,60,61,62,63,64,65,66,67,68,69,70,71,72,73,74,75,76,77,78,79,80,81,82,83,84,85,86,87,88,89,90,91,92,93,94,95,96,97,98,99,100,101,102,103,104,105,106,107,108,109,110,111].

The first and third most cited articles shown in Figure 2 studied nanocomposite scaffolds for bone tissue engineering. These scaffolds were produced by L-PBF of calcium phosphate /poly(hydroxybutyrate-co-hydroxyvalerate) (CaP/PHBV) and carbonated hydroxyapatite /poly(L-lactic acid) (CHAp/PLLA) polymer nanocomposite powder feedstocks [11,13]. The second and fifth most cited articles studied the effect of carbon-based additives as carbon nanofiber [12] and carbon black [15] in PA12 powder feedstocks. The fourth most cited article compared the fatigue properties of injection-molded and L-PBF-processed PA12 parts [14].

In the most cited article, a highly porous (>60%) cellular structure is produced by using microspheres of PHBV with 15 wt.% Ca-P and PLLA with 10 wt.% CHA. It was found that Ca-P nanoparticles improved cell proliferation and alkaline phosphatase activity of PHBV parts [11]. The second most cited article focused on producing 3 wt.% carbon nanofiber additivated PA12 powder feedstocks by melt mixing and cryogenic fracturing method. The carbon nanofiber additivated powder was processed by L-PBF, achieving a 22% increase of the storage modulus of PA12 with the addition of only a 3 wt.% of carbon nanofibers [12]. In the third most cited article, 15 wt.% Ca–P additivated PHBV microspheres were produced. After L-PBF processing, highly porous osteoconductive nanocomposite parts are built-in complex shapes [13]. The fourth most cited article compared the tensile properties of injection molded and L-PBF processed PA12 parts and found that L-PBF parts have almost the same tensile strengths and 15% higher elastic modulus compared to injection-molded ones [14]. Finally, in the fifth most cited article, adding a 4 wt.% of carbon black (CB) to PA12 and L-PBF processing of the additivated powder leads to a 20% decrease of the built part’s flexural modulus due to insufficient polymer–filler interface. However, the electrical conductivity is increased five orders of magnitude compared to as-built PA12 parts [15].

As a primary outcome of the five most cited publications, the additivation of polymer powder feedstock to achieve new powder compositions and enhance processability and as-built part properties are the most prominent trends over the last decade. These publications also indicate that researchers mostly use PA12 due to their ease of processability by L-PBF. From the application point of view, biocompatible or biodegradable polymer composite parts are growing in interest over the last decade.

The present study was further expanded to the 100 most cited articles in the following sections, finding a total of 257 polymer powder compositions [11,12,13,14,15,16,17,18,19,20,21,22,23,24,25,26,27,28,29,30,31,32,33,34,35,36,37,38,39,40,41,42,43,44,45,46,47,48,49,50,51,52,53,54,55,56,57,58,59,60,61,62,63,64,65,66,67,68,69,70,71,72,73,74,75,76,77,78,79,80,81,82,83,84,85,86,87,88,89,90,91,92,93,94,95,96,97,98,99,100,101,102,103,104,105,106,107,108,109,110,111]. The statistical evaluation performed is divided into three sections, the material, process, and as-built part properties. Each section’s most significant parameters are statistically investigated to determine the limits and average values of each reported property.

## 3. Material Properties of Polymer Powder Feedstocks

### 3.1. General Properties and Material Type

**Figure 3:**
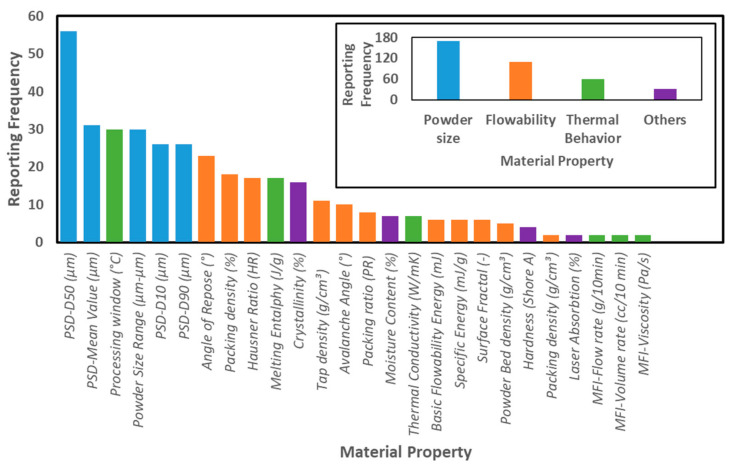
Reported material properties of polymer powder feedstocks and their reporting frequencies in the L-PBF articles [11,12,13,14,15,16,17,18,19,20,21,22,23,24,25,26,27,28,29,30,31,32,33,34,35,36,37,38,39,40,41,42,43,44,45,46,47,48,49,50,51,52,53,54,55,56,57,58,59,60,61,62,63,64,65,66,67,68,69,70,71,72,73,74,75,76,77,78,79,80,81,82,83,84,85,86,87,88,89,90,91,92,93,94,95,96,97,98,99,100,101,102,103,104,105,106,107,108,109,110,111]. The inset classifies the material properties into four general groups, powder size, flowability, thermal behavior, and others, defining the colors of the bars in the main graph.

L-PBF of polymer powder feedstocks depends significantly on the feedstock material’s properties and the parameters of the process. Hence, a statistical analysis is necessary to relate the feedstock polymer powder properties with the L-PBF processability, as-built part properties, and reproducibility of the results. To provide a clear view of the most reported material properties of polymer powder feedstocks, Figure 3 shows the reporting frequency for each of them. As observed in Figure 3, the D50 size value and mean size value are the most referred properties of the feedstock materials, provided 55 and 30 times, respectively. The inset in Figure 3 classifies the material characteristics into four general groups: powder size, flowability, thermal behavior, and others.

Values related to powder size and flowability are the most frequently provided properties in the evaluated publications, highlighting their relevance for L-PBF processing of the feedstock materials. To characterize the powder size and powder size distributions, D10, D50, D90, mean, and powder size range are reported, with the D50 value being most prominent. The angle of repose (AOR), packing density, and Hausner Ratio (HR) are the most significant parameters used in the articles to determine the flowability. The processing window, melting enthalpy, and thermal conductivity are the most reported material properties to determine the thermal behavior of the polymer powder feedstocks.

**Figure 4:**
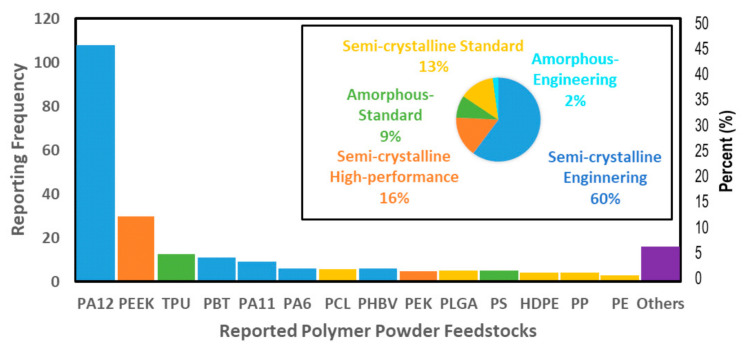
Reported polymer powder feedstocks processed by L-PBF. Others consist of POM, PU, DF, PA12-PEG blend, PA12-PVA blend, PEKK, PMMA, cellulose acetate, and starch-cellulose studied three times or less in the publications [11,12,13,14,15,16,17,18,19,20,21,22,23,24,25,26,27,28,29,30,31,32,33,34,35,36,37,38,39,40,41,42,43,44,45,46,47,48,49,50,51,52,53,54,55,56,57,58,59,60,61,62,63,64,65,66,67,68,69,70,71,72,73,74,75,76,77,78,79,80,81,82,83,84,85,86,87,88,89,90,91,92,93,94,95,96,97,98,99,100,101,102,103,104,105,106,107,108,109,110,111]. The inset shows the share of parent class polymers.

The polymers can be classified in terms of their melting temperature and crystalline structure to analyze further the materials employed for L-PBF. Depending on the melting temperature, the polymer powder feedstocks are classified as high-performance polymers (T_melting_ < 260 °C), engineering polymers (T_melting_ < 140 °C), and standard polymers (T_melting_ < 90 °C). Each sub-class can be divided into two groups depending on their molecular structure, i.e., amorphous or semi-crystalline [3,9]. According to this classification, the distribution of materials employed in the references [11,12,13,14,15,16,17,18,19,20,21,22,23,24,25,26,27,28,29,30,31,32,33,34,35,36,37,38,39,40,41,42,43,44,45,46,47,48,49,50,51,52,53,54,55,56,57,58,59,60,61,62,63,64,65,66,67,68,69,70,71,72,73,74,75,76,77,78,79,80,81,82,83,84,85,86,87,88,89,90,91,92,93,94,95,96,97,98,99,100,101,102,103,104,105,106,107,108,109,110,111] is displayed in Figure 4. The three most studied polymer materials, with a total frequency ranging from 17–107, are PA12, which is a semi-crystalline engineering polymer (S-E); PEEK, a semi-crystalline high-performance polymer (S-H); and TPU, an amorphous standard (A-S) polymer. The inset in Figure 4 shows the share according to the polymer powder group classification. The semi-crystalline engineering polymer powder feedstocks dominate with an overall presence of 60% in the evaluated references [11,12,13,14,15,16,17,18,19,20,21,22,23,24,25,26,27,28,29,30,31,32,33,34,35,36,37,38,39,40,41,42,43,44,45,46,47,48,49,50,51,52,53,54,55,56,57,58,59,60,61,62,63,64,65,66,67,68,69,70,71,72,73,74,75,76,77,78,79,80,81,82,83,84,85,86,87,88,89,90,91,92,93,94,95,96,97,98,99,100,101,102,103,104,105,106,107,108,109,110,111]. This group includes PA12, PBT, PA6, PA11, POM, and PLC. This value points at far more diverse use of polymer material types in research than in industry, as often a far higher L-PBF market share of 90–95% is reported even for PA12 alone [8,10].

### 3.2. Powder Size

**Figure 5:**
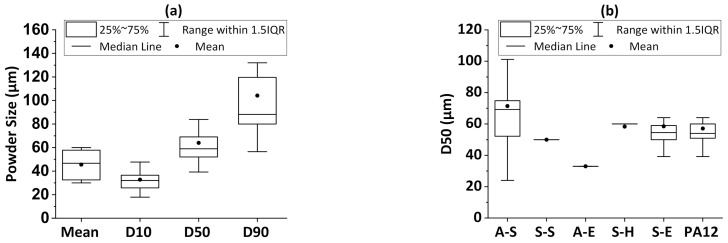
Range of reported polymer powder size characteristics: (**a**) statistics on the reported powder sizes as mean, D10, D50, and D90 values [11,12,13,14,15,16,17,18,19,20,21,22,23,24,25,26,27,28,29,30,31,32,33,34,35,36,37,38,39,40,41,42,43,44,45,46,47,48,49,50,51,52,53,54,55,56,57,58,59,60,61,62,63,64,65,66,67,68,69,70,71,72,73,74,75,76,77,78,79,80,81,82,83,84,85,86,87,88,89,90,91,92,93,94,95,96,97,98,99,100,101,102,103,104,105,106,107,108,109,110,111] (**b**) D50 values of polymer powders used in L-PBF of Amorphous Standard (A-S) including polymers such as TPU, PS, PMMA; of Semicrystalline Standard (S-S) including polymer as HDPE; Amorphous Engineering (A-E) including polymer as PDLLA; Semi-Crystalline High-performance (S-H) including polymers such as PEEK, PEK, PEKK; Semi-Crystalline Engineering (S-E) including polymers such as PBT, PA6, PA11, PA12, and PA12 powder feedstocks. Outlier points are not shown in plots.

The polymer classification based on their melting point is an approach to group them according to their thermal properties. However, a general processing procedure cannot be adopted for each polymer class due to the differences in composition, material properties, and inter-relationships. In that sense, the polymer powder size, shape, and additives affect the density [19,34,47], flowability [20,46], spreadability, and consequently, the processability of the material [20]. The powder layer’s minimum thickness, an important process parameter in L-PBF [71], is limited by the largest particles in the size distribution. The width of the powder feedstock size distribution also influences the L-PBF processability [20]. At least one of the statistical parameters such as the mean, D10, D50, or D90 value is typically provided in evaluated publications to characterize the powder size distribution. The reported values of these parameters in the references analyzed are plotted in Figure 5a. The D50 value was used in 54 studies, while the mean powder size was used in 8 studies, and the D10 and D90 values were used in 26 studies, reporting average values of 65 µm, 45 µm, 33 µm, and 108 µm, respectively [11,12,13,14,15,16,17,18,19,20,21,22,23,24,25,26,27,28,29,30,31,32,33,34,35,36,37,38,39,40,41,42,43,44,45,46,47,48,49,50,51,52,53,54,55,56,57,58,59,60,61,62,63,64,65,66,67,68,69,70,71,72,73,74,75,76,77,78,79,80,81,82,83,84,85,86,87,88,89,90,91,92,93,94,95,96,97,98,99,100,101,102,103,104,105,106,107,108,109,110,111]. Statistically, it was found that the D10 and D50 values have the narrowest range in the reported studies. A minimum D10 value of 18 µm was found for the TPU powder feedstocks [34]. As an outlier point for the D90 value, 389 µm was used for the 90 wt.% PBT/ 10 wt.% PC blend [58]. After this value, the maximum D90 value was 132 µm for the TPU powder feedstock [20]. As outlier points of the D50 value, 101 µm [20], 112 µm [68], 162 µm [58], and 200 µm [26] were studied. The min and max D50 values were 24 µm [41], which is an outlier point in Figure 5a, and 84 µm, respectively. A detailed analysis of the extracted D50 values for the different polymer’s classification groups and separately the PA12, as the most employed powder, are plotted in Figure 5b. The number of references where D50 values were found for each polymer class was 18 for A-S polymers, 6 for S-H polymers, 25 for S-E polymers, and 19 for PA12. The average D50 values for A-S, S-E, S-H, and PA12 varied between 58 µm and 71 µm. The highest deviation is found for the A-S polymers, varying between 24 µm [41] and 101 µm [20] with a distant point of 200 µm [26]. Only one data point was obtained for A-E polymers, related to a 50 wt.% Ca-phosphate/PDLLA, with a D50 value of 33 µm [98]. Overall, it is found that reported D90, D50, and D10 values of polymer powder feedstocks are below 130 µm, 80 µm, and 50 µm, respectively. The statistically narrow material-specific powder size values point at the awareness of employing defined particle size distributions over L-PBF processing. It is known that size distribution affects optimal processing parameters directly, spatial resolution, and the as-built part density. To assess such cross-influences of the particle size distribution, in the last section of this study, D50 values of polymer powder feedstocks will be evaluated by a principal component analysis to obtain further correlations with the process and as-built part properties.

The most reported material properties coming second and third after the powder size distribution are the powder flowability and the processing/sintering window [20,24,30,46,50,51,67,71,73]. A good flowability increases the density of the powder bed, which results in denser as-built parts. The flowability of powder feedstocks depends on the adhesion force between particles, depending on the powder geometry and surface roughness [112]. The adhesion between powders can be hindered by coating the surface of the polymer powder feedstocks with suitable nanoparticles [23,43,54,106]. Depending on the nanoparticle size, the amount needed to form a monolayer on the micropowder varies. It has been shown for 5 nm nanoparticles that the addition of 0.1 vol% can already form a monolayer over a PA12 powder having a D50 of 58 μm and a specific surface area of 0.1 m^2^/g [113]. These nanoparticles should be homogeneously distributed on the surface to maximize their effects at given vol%. Consequently, aggregations should be avoided. Only a defined surface roughness and nanoparticle coverage may create glidant effects.

### 3.3. Flowability and Processing Window

**Figure 6:**
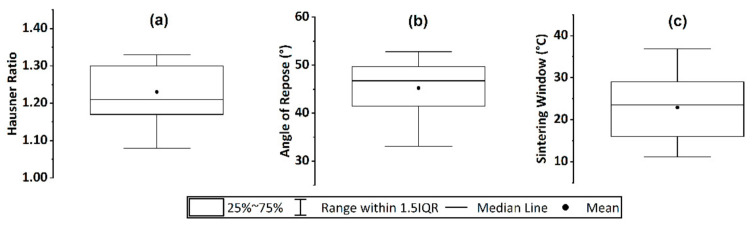
Statistics on flowability and sintering parameters of (**a**) Hausner Ratio and (**b**) angle of repose reported for flowability behavior, and (**c**) sintering window reported for thermal behavior of polymer powder feedstocks.

The flowability and processing window plays an essential role in the L-PBF process and the manufactured parts’ properties. Consequently, to evaluate the powders employed in the analyzed references, the Hausner ratio (HR), the angle of repose (AOR), and the sintering window values are extracted. The analysis shown in Figure 6 displays the 17 values of the HR, 20 values of the AOR, and 32 values of sintering windows that are reported. A high flowability is obtained if the HR value is below 1.25, and cohesive behavior that avoids powder flow is considered if the HR is above 1.4 [20,46,51]. As seen in Figure 6a, HR values vary between 1.08 and 1.33 with an average value of 1.23. Values higher than 1.25 are only found for two of the references analyzed, PBT and POM [46,51]. Other HR values below 1.25 are obtained for TPU, PP, POM, PE, PA6, and PA12 powder feedstocks [20,46,51]. The other important parameter standardly measured to evaluate the flowability is the AOR. The lower the AOR, the higher the flowability [50,65]. The reported AOR values vary between 33° for PA12 powder feedstocks [50] and 53° for PEEK powder feedstocks, with an average value of 45° [51,65]. The polymer powder L-PBF process temperature depends on the gap between the melting and the crystallization temperature of the polymer material, i.e., a meta-stable thermodynamic region defined as “sintering window” [8]. The sintering window reported values are shown in Figure 6c, ranging from 11 °C for PA11 powder feedstock [67] to 37 °C for PA6 polymer feedstock [71], with an average value of 23 °C. In conclusion, according to the reported HR and AOR values, it is expected that good flowabilities result in higher powder bed densities with better processability and as-built part property (see Section 6). It can be stated that the sintering window of the same polymer class, i.e., PA12, highly depends on its molecular weight, crystallinity, and aging degree after several process cycles. Hence, the control of those parameters is required to ensure the repeatability of the L-PBF process and as-built parts properties.

### 3.4. Additives

**Figure 7:**
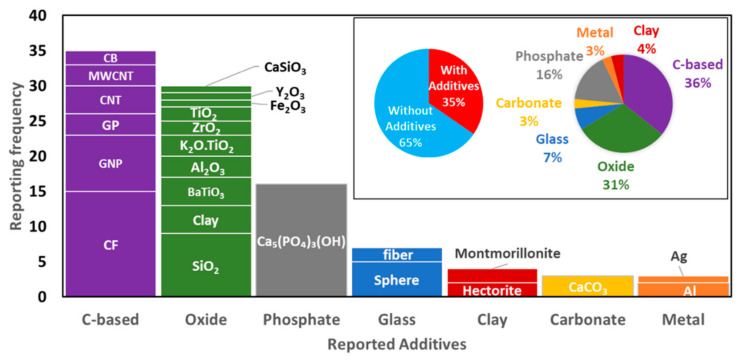
Bibliometrics of additive material classes and subtypes. The inset shows the overall share of additives within reported powder compositions and the additive classes percentage.

Incorporating additives to increase the processability of a powder or to enhance and/or add functional properties to the as-built parts is a widely used approach. From the evaluated references, additives were used in 94 polymer powder compositions, comprising 36% of all reported powder compositions, as shown in the inset of Figure 7.

Additives have been reported to change the crystallization kinetics during cooling [27,36], induce heterogeneous nucleation [18,26], increase laser absorption [32,61], enhance mechanical properties [12,16,22,28,29,39,48,65], and even provide new functional properties [15,21,25,38,67,74]. Several materials have been proposed as additives due to the wide range of properties desired to modify the polymer built parts for different applications. In Figure 7, the reported additives are grouped by material classes, finding the presence of C-based ones (carbon fiber (CF), graphene nanoplatelets (GNP), graphene platelets (GP), carbon nanotube (CNT), multi-wall carbon nano tube MWCNT, and carbon black (CB)) [21,22,23,24,25,26,27,28,29,30,38,39,45,47,61,65,77,78,89,101], oxides (SiO_2_, BaTiO_3_, Al_2_O_3_, K_2_O.TiO_2_, ZrO_2_, TiO_2_, Fe_2_O_3_, Y_2_O_3_, and CaSiO_3_) [23,24,28,67,79,80,83,90,105,106,108,110], clays (sulfonated montmorillonite, organo-modified montmorillonite, and hectorite) [25,31,36], glass [32,48,100], phosphate (hydroxyapatite and Ca-phosphate) [13,50,74,81,82,86,97,98], carbonate (CaCO_3_) [50], and metals (Al, Ag) [24,94]. A quantitative analysis of the data shows that C-based additives are the most studied ones with a share of 37%. These additives are mostly used to enhance absorbance, mechanical properties, and/or electrical conductivity of the built parts. The C-based additives are followed by oxides with a share of 28%. Next come the phosphates with a share of 17% addressing in biomedical applications as bone tissue engineering. Within each of the additives’ general groups, carbon fiber (CF) and Ca-phosphate are the most studied additives in L-PBF of polymer powder feedstocks, i.e., 15 studies mixing CF with PA12 powder feedstock [12,16,29,39,61] and PEEK powder feedstock [47], and 16 studies mixing Ca-phosphate with PHBV [13,81,82,97], PDLLA [98], PA12 [102], PLGA [107], and PCL [86], with high weight loads varying between 5 and 50 wt.%.

To sum up, incorporating different additives into the polymer powder feedstocks is a quite frequent approach to increase the built parts’ processability, biocompatibility, mechanical, thermal, or electrical properties. It should be noted that future developments on micro- or nano-additivation of the polymer powder feedstocks exhibit a high potential to develop new powder feedstock for L-PBF of polymers and built parts with enhanced performance and new functionalities [114,115,116].

## 4. L-PBF Machine and Process Parameters

**Figure 8:**
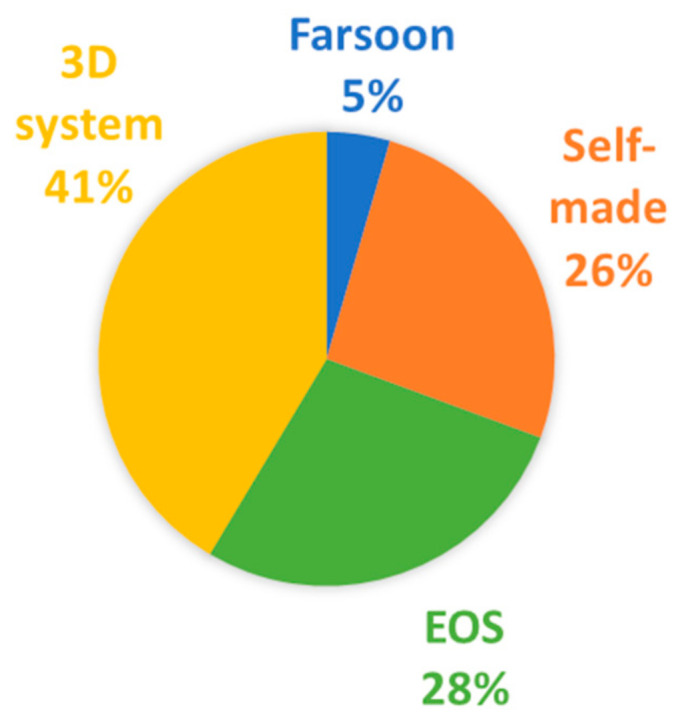
Share of L-PBF machine manufacturers reported for L-PBF of polymer powder feedstocks in the studies [11,12,13,14,15,16,17,18,19,20,21,22,23,24,25,26,27,28,29,30,31,32,33,34,35,36,37,38,39,40,41,42,43,44,45,46,47,48,49,50,51,52,53,54,55,56,57,58,59,60,61,62,63,64,65,66,67,68,69,70,71,72,73,74,75,76,77,78,79,80,81,82,83,84,85,86,87,88,89,90,91,92,93,94,95,96,97,98,99,100,101,102,103,104,105,106,107,108,109,110,111].

In the previous section, it was shown how the material properties of the different types of polymer powder feedstocks can vary in their reporting frequencies. However, the polymer powder properties influence the L-PBF manufacturing process, and the system parameters of the L-PBF process are also crucial factors to consider. Several L-PBF machine manufacturers exist, and each commercial machine’s different options may play a role in the L-PBF manufactured parts. A statistical analysis of the employed L-PBF machines is performed in Figure 8 to provide a view of the systems used in research. In 60% of the evaluated references, the researchers reported L-PBF machine manufacturer [11,12,13,14,15,16,17,18,19,20,21,22,23,24,25,26,27,28,29,30,31,32,33,34,35,36,37,38,39,40,41,42,43,44,45,46,47,48,49,50,51,52,53,54,55,56,57,58,59,60,61,62,63,64,65,66,67,68,69,70,71,72,73,74,75,76,77,78,79,80,81,82,83,84,85,86,87,88,89,90,91,92,93,94,95,96,97,98,99,100,101,102,103,104,105,106,107,108,109,110,111]. 3D Systems is the principal provider of L-PBF machines for processing polymer powders, with a 41% share, followed by EOS with a 28% share, and Farsoon with a 5% share. It should be noted that 26% of the L-PBF machines are self-made. The machine types and the variability between them and operators may be expected to affect processing and as-built part property variations. To control that, round-robin or inter-laboratory studies for L-PBF of metal powders study the variation in the mechanical properties of as-built parts between study partners, proving that it is much higher than the variability between the parts produced by the same user [117,118,119]. Consequently, round-robin studies for L-PBF of polymer powder feedstocks represent a promising approach to better understand the determinants of repeatability and reproducibility of the process and further control the factors that affect them.

**Figure 9:**
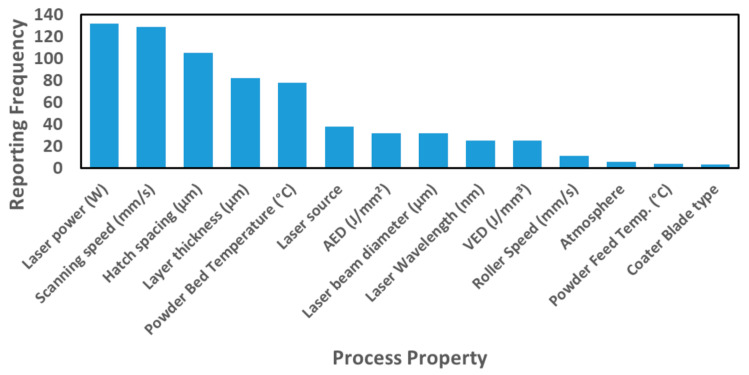
Reported process properties for L-PBF of polymer powder feedstocks and their reporting frequencies in the articles [11,12,13,14,15,16,17,18,19,20,21,22,23,24,25,26,27,28,29,30,31,32,33,34,35,36,37,38,39,40,41,42,43,44,45,46,47,48,49,50,51,52,53,54,55,56,57,58,59,60,61,62,63,64,65,66,67,68,69,70,71,72,73,74,75,76,77,78,79,80,81,82,83,84,85,86,87,88,89,90,91,92,93,94,95,96,97,98,99,100,101,102,103,104,105,106,107,108,109,110,111].

As mentioned, the features and parameters of the different machines influence the L-PBF outcome. The reported process parameters are given in Figure 9, with the four most reported process parameters being laser power, scanning speed, hatch spacing, and powder layer thickness [11,12,13,14,15,16,17,18,19,20,21,22,23,24,25,26,27,28,29,30,31,32,33,34,35,36,37,38,39,40,41,42,43,44,45,46,47,48,49,50,51,52,53,54,55,56,57,58,59,60,61,62,63,64,65,66,67,68,69,70,71,72,73,74,75,76,77,78,79,80,81,82,83,84,85,86,87,88,89,90,91,92,93,94,95,96,97,98,99,100,101,102,103,104,105,106,107,108,109,110,111].

Even though the parameters may seem unconnected as they refer to the laser properties, powder bed dimensions, and scanning system, the volumetric energy density (VED), Equation (1), relates them and is often used as a first approach to compare different systems:

VED = P/v·h·t (J/mm^3^) (1)

where P is the laser power applied (W), h is the hatch distance (mm), t is the thickness of the powder layer (mm), and v is the scanning speed (mm/s).

The areal energy density (AED) removes the dependence with the powder layer thickness and is also a useful property to understand powder bonding, inter-particle diffusion, and the melting–cooling mechanism [37,53]. AER can be calculated from Equation (2):

AED = P/v·h (J/mm^2^) (2)

While the VED and AED are good indicators of the similarity between experimental setups in L-PBF, for a complete reproducibility of the process, the individual parameters should be matched as each parameter can individually affect, for example, the density or the mechanical properties of the built part. Bourell et al. [120] investigated the correlation of density and mechanical strength of PA12 parts with several energy density formulations such as linear (J/mm), areal (J/mm^2^), and volume (J/mm^3^). They focused on integrating laser beam diameter as an inversely proportional function in volume-based energy density and found that the VED provides the best correlation with final part density and mechanical strength.

The different parameters of the L-PBF process are highly entangled, and the modification of one of them often affects the others. Consequently, it is interesting to study each parameter’s variation to provide a window of optimum values as a reference for future studies. In this way, researchers used several process parameters to obtain the highest relative densities, or the highest mechanical properties of as-built parts in the references studied.

**Figure 10:**
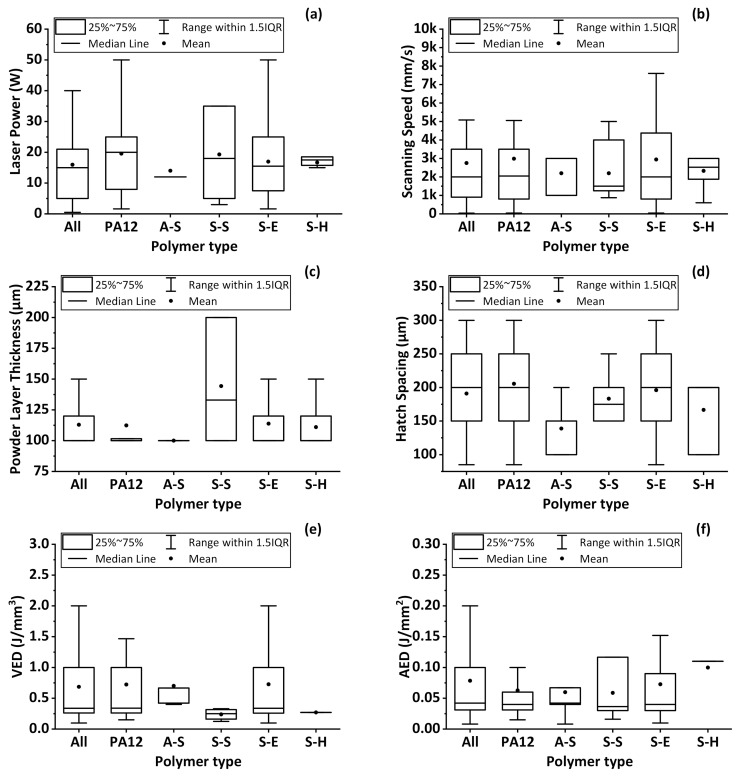
Bibliometrics of the most reported L-PBF process parameters: (**a**) laser power, (**b**) scanning speed, (**c**) powder layer thickness, (**d**) hatch spacing, calculated (**e**) volumetric energy density, and (**f**) areal energy density. The study differentiates between all polymers, PA12, A-S, S-S, S-E, and S-H powder feedstocks [11,12,13,14,15,16,17,18,19,20,21,22,23,24,25,26,27,28,29,30,31,32,33,34,35,36,37,38,39,40,41,42,43,44,45,46,47,48,49,50,51,52,53,54,55,56,57,58,59,60,61,62,63,64,65,66,67,68,69,70,71,72,73,74,75,76,77,78,79,80,81,82,83,84,85,86,87,88,89,90,91,92,93,94,95,96,97,98,99,100,101,102,103,104,105,106,107,108,109,110,111]. Outlier points are not shown.

The process parameters required to obtain the highest as-built part properties [11,12,13,14,15,16,17,18,19,20,21,22,23,24,25,26,27,28,29,30,31,32,33,34,35,36,37,38,39,40,41,42,43,44,45,46,47,48,49,50,51,52,53,54,55,56,57,58,59,60,61,62,63,64,65,66,67,68,69,70,71,72,73,74,75,76,77,78,79,80,81,82,83,84,85,86,87,88,89,90,91,92,93,94,95,96,97,98,99,100,101,102,103,104,105,106,107,108,109,110,111] were extracted for 257 powder compositions. Laser power, scanning speed, powder layer thickness, hatch spacing, calculated VED, and calculated AED values, which were obtained in 149, 146, 98, 119, 62, and 118 studies, respectively, are plotted in Figure 10a–f. Furthermore, the optimized values of process parameters for the most studied polymer type PA12 and the other polymer type classes (A-S, S-S, S-E, and S-H) to obtain the highest density or the highest mechanical properties are also provided in Figure 10a–f.

Reported laser powers, which are in direct proportion to energy density, are shown in Figure 10a. The lowest laser power of 2 W was used for PA12 [56] and cellulose acetate [91], while the highest laser power of 50 W was used for PA12 with areal energy densities varying between 0.02 and 0.1 J/mm^2^ [53]. The narrowest dispersion of laser power values was obtained for A-S polymers, specifically TPU [20,35], PU, and MWCNT-PU [30] powder feedstocks. A wide value scattering and higher average laser power of 19W was obtained for S-S polymers like HDPE [78] and PP [51,64,79,80] powder feedstocks. S-E polymers followed a similar trend as PA12. S-H polymers such as PE [92], PEEK [47,66,77], PEK [96], and PEKK [56] exhibit a narrow dispersion of the laser power values. It was found that different polymer types have been processed with laser power values ranging from 2 W to 50 W. However, the average laser power for A-S, S-S, S-E, and S-H polymers is 12 W, 18 W, 16 W, and 15 W, with an average processing value for all polymer classes of 16 W.

The scanning speed, which affects the VED and the total duration of the build cycle, is evaluated in Figure 10b. Scanning speeds varied in a broad range to process all types of polymer feedstocks. The lowest scanning speed of 45 mm/s was used for PA12/MWCNT composite powders [21], and the fastest scanning speed of 12100 mm/s (outlier point) was used for Al and AlCuFeB additivated PA12 powders [94]. A-S, S-S, and S-H polymer types have almost the same average scanning speed value of 2500 mm/s. The highest scanning speeds for A-S polymers was 3000 mm/s, employed to process MWCNT-PU powders [30], while for S-S polymers, 5000 mm/s were used for PP powders [51]; and for S-H polymers, 3000 mm/s was employed to process carbon fiber-PEEK powders [47]. PA12 powder feedstocks followed the same trend as the rest of the polymer powder feedstocks.

The powder layer thickness, Figure 10c, varied between 100 µm and 200 µm for all powder feedstocks. The narrowest value distribution was obtained for PA12 and A-S polymers. The thickest powder layer of 200 µm was used to process Al_2_O_3_-PP [80], PA12 [87], and MWCNT-PA12 powders [21]. It was found that reducing powder layer thickness below 150 µm is a trend in processing, keeping good processability of the powders with the given material properties.

Analyzing the hatch spacing, Figure 10d, the parameter varied between 85 µm and 300 µm. The lowest hatch spacing of 85 µm was used for Ca-phosphate-PA12 powders [102]. As an outlier point, the highest value of 700 µm was used for PA12 powders [53]. Additionally, hatch spacing of 300 µm was used for PA12/TiO_2_ and PA12/GNP composites with an AED of 0.06 J/mm^2^ [28], PA12 with an AED of 0.03 J/mm^2^ [88,89,90], PA12/CNT composite with an AED of 0.04 J/mm^2^ [45], and PA6 and PA12 with an AED of 0.02 J/mm^2^ [71]. Setting a hatch spacing below 300 µm is a general value trend in processing different polymer types for the given powder size ranges. The distance of hatch spacing can also be linked to the laser beam diameter. Several studies reported a hatch distance, which was mostly half of the laser beam diameter. With this result, we can estimate a trend on using a laser beam diameter below 600 µm to process different types of polymers where the average D50 value was 80 µm.

The variation in the individual parameters mentioned above is transferred to the VED and AED. The calculated VED, Figure 10e, ranged from 0.1 to 2 J/mm^3^, with an average value of 0.7 J/mm^3^ to process studied polymer compositions. PA12 and S-E polymer types have a wider dispersion of the VED values. As outlier points, 3.6 J/mm^3^ were employed for PA12 powders [87], and 3.4 J/mm^3^ were used for MWCNT-PA12 powders [21]. For the individual polymer classes, the highest calculated VED employed to process TPU powder was 1.6 J/mm^3^ [35]. Only one data point was extracted for S-H polymer powder (PEEK) [66]. Except for the outlier points, a VED lower than 2 J/mm^3^ and VED median value well below 0.5 J/mm^3^ is used to process all types of polymer powder feedstocks.

Finally, the AED, Figure 10f, ranged from 0.01 to 0.2 J/mm^2^ for all polymers. The maximum value employed was 0.7 J/mm^2^ for PA12 powders [87]. As a general value trend, mean <0.1 J/mm^2^ and median around 0.05 J/mm^2^ of AED is used to process all polymer types.

Overall, the “virtual mean” process parameter set of polymer L-PBF is processed with the mean laser power of 16W, scanning speed of 2800 mm/s, powder layer thickness of 110 µm, hatch spacing of 190 µm, VED of 0.7 J/mm^3^, and AED of 0.08 J/mm^2^.

## 5. As-Built Part Properties of Polymer Powder Feedstocks

**Figure 11:**
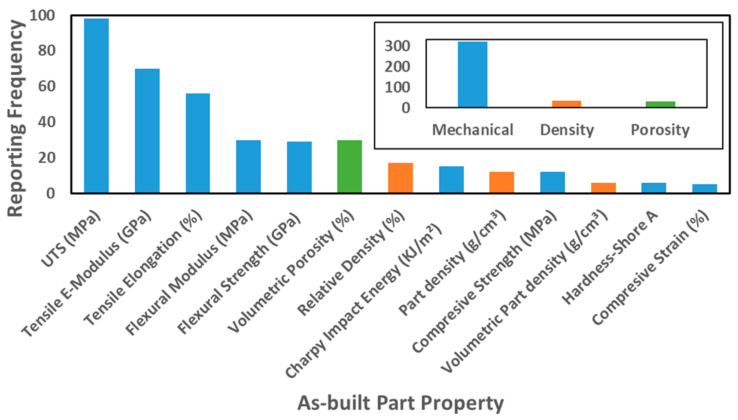
Reported as-built part properties of polymer powder feedstocks processed by L-PBF and their reporting frequencies in the studies [11,12,13,14,15,16,17,18,19,20,21,22,23,24,25,26,27,28,29,30,31,32,33,34,35,36,37,38,39,40,41,42,43,44,45,46,47,48,49,50,51,52,53,54,55,56,57,58,59,60,61,62,63,64,65,66,67,68,69,70,71,72,73,74,75,76,77,78,79,80,81,82,83,84,85,86,87,88,89,90,91,92,93,94,95,96,97,98,99,100,101,102,103,104,105,106,107,108,109,110,111]. The inset shows the grouped sum of the reported as-built part properties, defining the colors of the bars in the main diagram.

After addressing the material properties and processing parameters the resulting most relevant as-built part properties are studied. However, first, the principal parameters employed to evaluate the as-built part properties are extracted and individually analyzed. As seen in Figure 11, the most investigated part property was the ultimate tensile strength (UTS), which is reported 100 times within the references studied. Tensile and flexural tests are the most reported techniques to evaluate mechanical properties. The inset in Figure 11 shows that mechanical properties, which are reported around 300 times, are addressed far beyond other as-built part properties, such as density and porosity.

**Figure 12:**
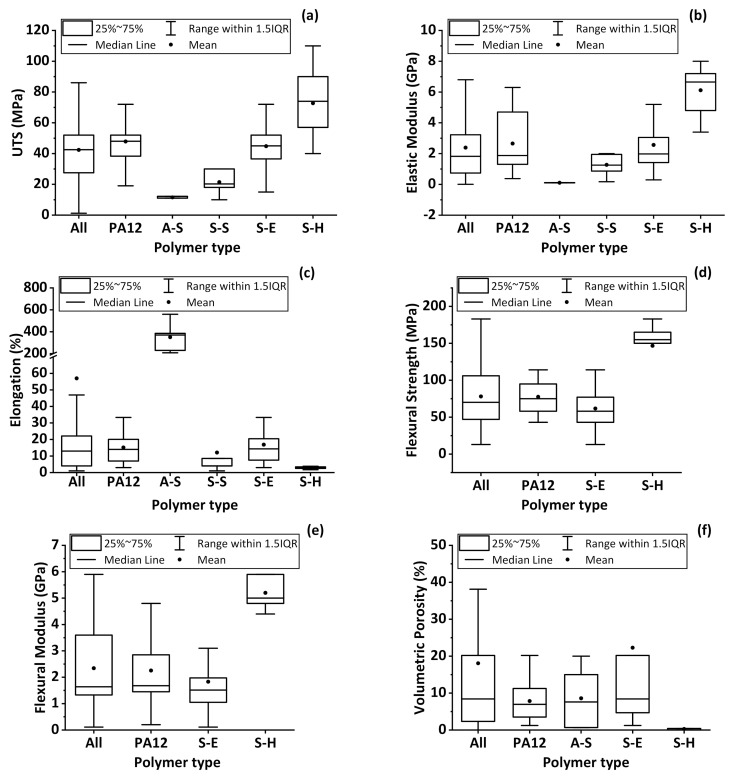
Statistics of the most reported as-built part properties of all polymers, PA12, A-S, S-S, S-E, and S-H powder feedstocks processed by L-PBF process: (**a**) ultimate tensile strength, (**b**) elastic modulus, (**c**) elongation, (**d**) flexural strength, (**e**) flexural modulus, and (**f**) volumetric porosity. Outlier points are not shown.

The statistics of resulting tensile test values (Figure 12a–c), flexural test values (Figure 12d–e), and volumetric porosity values (Figure 12f) for as-built, PA12, A-S, S-S, S-E, and S-H polymer parts are shown in Figure 12 to provide a detailed view of the properties of each built part. It should be noted that, since there are no available standards developed for testing tensile and flexural properties of L-PBF polymer parts, different specimen dimensions, geometries, and testing conditions between the evaluated references may result in different final part properties for the same polymer type produced under same L-PBF process conditions.

On the other hand, the available standards are developed for testing moulded and extruded polymer parts having almost 100% relative density and high ductility. Most of the L-PBF polymer parts do not show such a high relative density, and their ductility is lower compared to moulded and extruded ones.

As can be seen in Figure 12a, the average value of the UTS was 42 MPa for all polymers, with a minimum value of 1.2 MPa for cellulose acetate parts [91], and as an outlier point, a maximum value of 110 MPa for a PEEK/10 wt.% CF composite powder feedstock [48]. Depending on the process parameter sets and the additives used, the UTS of PA12 parts varies between 20 and 70 MPa. As an outlier point of PA12 values, 94 MPa was obtained for a 0.5 wt.% MWCNT-PA12 part [21]. The lowest average UTS is obtained for A-S polymer types, and TPU parts exhibit the highest value of 18 MPa within the UTS values of A-S polymer types [20,35]. For S-S polymers, an average value of 20 MPa with a peak value of 30 MPa is obtained in PP parts [44,64]. For S-E polymers, an average value of 45 MPa, with the highest value of 94 MPa, is reported for a 0.5 wt.% MWCNT-PA12 part [21]. S-H polymers have an average value of 75 MPa with the highest value of 110 MPa obtained in a 10 wt.% CF-PEEK part [47]. The built parts from different polymer classes show increasing UTS value in order as A-S < S-S < S-E < S-H. The employment of (carbon-based) additives increased the UTS of polymer parts up to 50%.

The elastic modulus (EM) analysis results, Figure 12b, reveal a high dispersion of the values when all polymer types are evaluated. The lowest reported value is 0.004 GPa for an elastomer [20] and the highest is 8 GPA for 15 wt.% CF-PEEK parts [47]. EM of PA12 parts varies between 0.37 GPa for a PA12 part [71] and 6.3 GPa for a CF-PA12 part [121]. Similar to UTS values (Figure 12a), the EM of the as-built parts of the polymer classes increase in the order A-S < S-S < S-E < S-H. A-S polymer types have an average EM value of 0.1 GPa with the highest value of 0.12 GPa found for TPU parts [20]. S-S polymer types exhibit an average EM value of 1.1 GPa with a peak value of 1.25 GPa obtained for HDPE parts [44]. The average EM of S-E polymer type is 2.6 GPa, with the highest value of 6.3 GPa obtained in the CF-PA12 part [121]. The S-H polymers have an average EM of 6 GPa, with the highest value of 8 GPa reported for 15 wt.% CF-PEEK parts [47]. Similar to UTS, carbon-based additives enhanced the elastic modulus of polymer parts.

The elongation results of the reported built parts are summed up in Figure 12c. The elongation during the tensile test varies from a minimum value of 1% for 20 wt.% PA12-PP blend to a maximum one of 560% for TPU [35]. The polymer classes’ analysis shows that the elongation of PA12 parts ranges from 3% [90] to 47% [71]. In addition, due to elastomers’ nature, A-S polymers resulted in the highest elongations with an average elongation of 380% and a maximum of 560% for a TPU part [35]. The average value for S-S polymers is 12%, and a maximum of 43%, an outlier point in Figure 12c, is reported for a PE part [104]. The S-E polymer class exhibits an average elongation of almost 20% and 76% maximum and a maximum outlier point of 76% for PA6 parts [71]. For the S-H polymer type, an average value of 3% is obtained, and a maximum of 3.8% for PEK parts [96].

The flexural strength (FS), Figure 12d, shows a minimum of 13 MPa obtained for 50 wt.% PA6 and 50 wt.% PA12 blend [75] and a maximum of 183 MPa for a 5 wt.% CF-PEEK part [47]. The PA12 parts show an average FS of 80 MPa and 114 MPa for oxidized heat-treated CF-PA12 parts as the highest value for PA12 and S-E polymers [29]. The average FS for S-E polymer is 70 MPa. For the S-H class, the average FS is 150 MPa, and the highest value of 183 MPa is obtained for 5 wt.% CF-PEEK parts [47]. No FS data have been reported for A-S and S-S polymer types.

The other property obtained in flexural tests is the flexural modulus (FM). Scattering of the FM can be seen in Figure 12e. Values are reported between 0.11 GPa obtained in PA6-20 wt.% PA12 blend [75] and 5.9 GPa obtained in 15 wt.% CF-PEEK parts [47]. In PA12 parts, the average FM is 2.2 GPa, while the highest value of 5.9 GPa is obtained for CF-PA12 parts [47]. The S-E and S-H polymer types have an average FM of 1.7 GPa and 5.5 GPa, respectively. The maximum FM for S-H is 5.9 GPa obtained in 15 wt.% CF-PEEK parts [47]. No data have been found for the FM of A-S and S-S polymer types.

As a common remark on the evaluated mechanical properties, the polymer parts with carbon-based additives exhibited the highest mechanical strengths and modules in the evaluation. Since an increase in mechanical strength is often linked to decreased porosity content in sintered parts, the volumetric porosity (VP) values are extracted from the references to confirm this trend (Figure 12f) in the following.

Volumetric porosities varied between 0.01%, reported for GP-PEEK [65], and as high as 38%, reported for an elastomer part [20]. The PA12 parts have an average VP of 9%, and maximum densification is achieved with a VP of 1.2% obtained in 1 wt.% MWCNT-PA12 part [30]. For the A-S polymer, the average VP is 10%, and the maximum densification is achieved with a VP of 0.6% for 1 wt.% MWCNT-PU parts [30]. S-E polymer types have an average VP of 20%, and densification is maximized with a VP of 1.2% [30]. A narrow distribution is obtained for S-H class, with an average VP of 0.05% and maximum densification for a VP of 0.01% reported for GP-PEEK parts [65].

Overall, the presence of carbon-based additives in most of the built parts with best performance for each property confirms that these additives are significant. Still, high loads of additives often increased the volumetric porosity, which can be linked to non-optimized wetting between additives and polymer matrix during processing. High volume loadings of additives, often employed if mechanical properties are intended to be improved, further complicate their good dispersion [13]. It has been proved for CF-polymer composites that a homogeneous distribution of the fibers in the matrix with a high fiber aspect ratio and strong adhesion to polymer matrix provide the highest mechanical properties [122]. In addition to the effect of carbon fibers on the mechanical properties of polymer parts, there are different fiber materials that can be used as an additive in polymer matrix to add functional properties to the L-PBF generated parts [123]. The dispersion may be improved by adding matrix-compatibilization chemicals [26,124]. Note that carbon nanoparticle deposited on polymer powders already has an effect at very low weight loadings of 0.005 vol%, affecting crystallization orientation during L-PBF, as has been recently shown by Sommereyns et al. [125]. The lowest mechanical strengths and elastic modulus with the highest ductility are obtained for amorphous standard polymers. In contrast, the semi-crystalline high-performance polymers exhibit the highest mechanical strength and elastic modulus but the lowest ductility. These S-H polymers also resulted in the highest densifications of over 99%. Since S-H polymers exhibit the highest mechanical responses, it is found that the UTS of PA12 powders using additives can be enhanced to the average UTS of S-H polymers, which can open new application fields for PA12 in the polymer industry that uses S-H polymers. Nevertheless, this is not the case for flexural strength. S-H polymers’ flexural strengths are still far higher than S-E polymers and still the best industrial candidates when the parts are used under flexural deformations.

In this context, the average mechanical properties of PA12, the most prominently used polymer, resulted in an UTS of 48 MPa, EM of 2.8 GPa, elongation of 16%, FS of 76 MPa, and FM of 2.4 GPa, where the average volumetric porosity is 8%. Moreover, the average mechanical properties of PEEK, the most robust used polymer, resulted in a UTS of 75 MPa, EM of 6.3 GPa, elongation of 4%, FS of 150 MPa, FM of 5.4 GPa, where the average volumetric porosity was 1%.

## 6. Quantification of Cross-Correlations by Principle Component Analysis of the Most Reported Material, Process, and as-Built Part Properties

A Principle Component Analysis [126,127] is used to assess the correlation between reported material, process, and as-built part properties. A comprehensive data matrix was extracted from the references. A total of 257 polymer powder compositions are used as observation labels, and the most reported properties (see Figure 3, Figure 9 and Figure 11) are used as variables in the PCA matrix. It was possible to analyze eight powder compositions and their related properties by PCA, as only for those eight polymers all nine variables required for the analysis have been reported [21,27,35,70,71].

**Figure 13:**
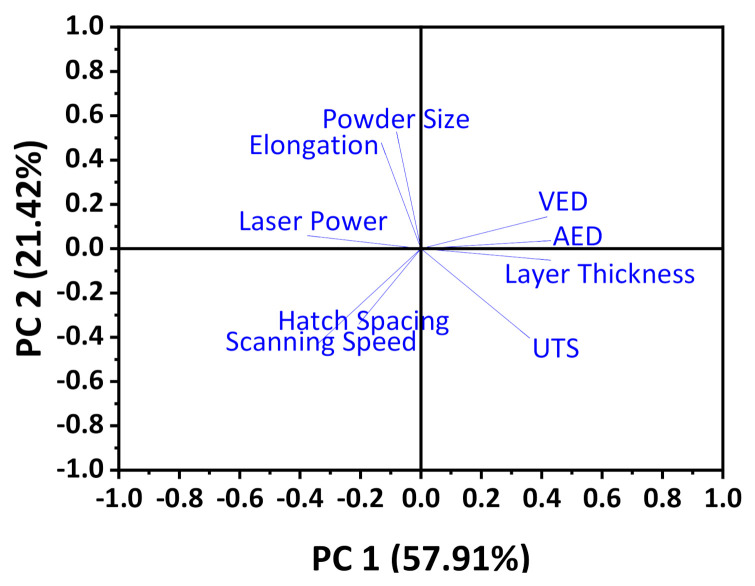
Principal component analysis 2D loading plot of various material, process, and as-built part properties.

PCA statistically evaluates the correlation between variables such as powder size (including D50 and mean size), laser power, scanning speed, hatch spacing, the powder layer thickness, VED, AED, UTS, and elongation. The results summarized in Figure 13 from the PCA analysis evidence that components 1 and 2 (PC1 and PC2) represent 58% and 21% of the inter-components variation. Moreover, the different experimental parameters analyzed in the PCA are represented in Figure 13, highlighting the existing relations between them and the subspace dimensions. The results arising from that analysis proves that VED, AED, powder layer thickness, and laser power are the most influencing parameters on the PC1, while powder size and elongation are the parameters that influence PC2 the most. The relationship between the slopes of the linear dependencies found for each experimental parameter with the principal components can be used to calculate their correlations. The differences in the slopes, and therefore the correlation between variables, can be also quantified by the angles formed between the linear fits of the specific parameters that need to be evaluated. These correlations directly show the influence of each of the parameters over the overall L-PBF process.

The correlation matrix obtained from the PCA is quantified in Table 1. Negative values indicate a negative correlation between the properties. As expected, a positive correlation value of 0.98 is found between VED and AED, two physically closely connected parameters. Interestingly, the second-highest positive correlation, 0.85, is found between UTS and powder layer thickness. Next, the UTS and calculated AED correlate by 0.78, while the scanning speed and hatch spacing correlation value is 0.72. In the case of powder size and laser power, the value is 0.40. On the other extreme, a negative correlation is obtained between reported powder layer thickness and used laser power, −0.86.

**Figure 14:**
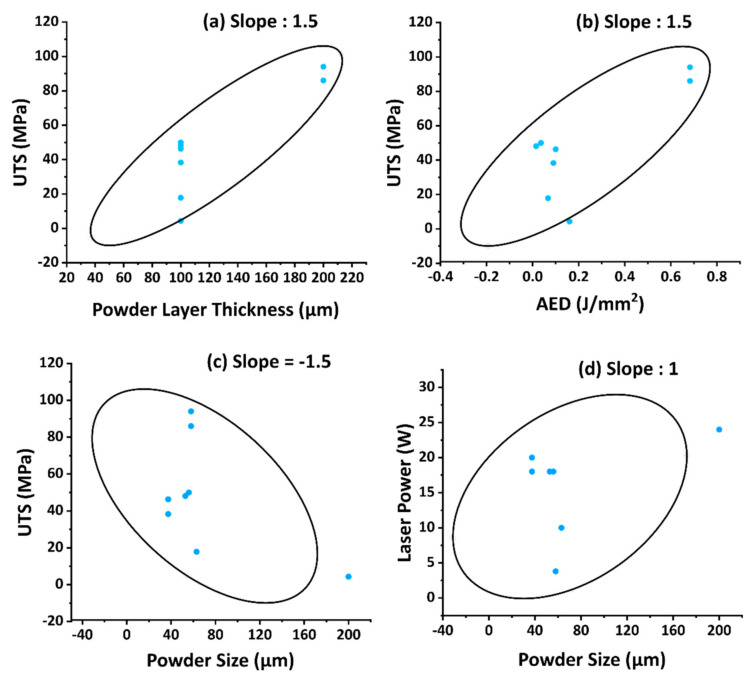
Scatter plots for (**a**) powder layer thickness vs. UTS, (**b**) AED vs. UTS, (**c**) powder size vs. UTS, and (**d**) powder size vs. laser power according to Table 1. A 68% confidence ellipse is depicted for each plot.

From the PCA correlation matrix given in Table 1, the highly correlated parameters, powder layer thickness vs. UTS (Figure 14a), AED vs. UTS (Figure 14b), powder size vs. UTS (Figure 14c), and powder size vs. laser power (Figure 14d) are plotted for the eight powder compositions extracted from the PCA data matrix. An ellipse indicating the 68% confidence level area is included in Figure 14a–d.

As shown in Figure 14, positive correlation trends are evidenced in Figure 14a,b,d, and a negative correlation trend is found in Figure 14c. The slope of the tangent line to the co-vertices of the ellipse in Figure 14a–d helps to understand the degree of correlation between the properties. The PCA analysis data matrix is shown in Table 2 to better understand the correlations between variables and powder compositions. The PCA results can be interpreted as follows: increasing the powder layer thickness by a factor of 2 would increase the UTS by a factor of 3, Figure 14a. This PCA result is dominated by a study using a too high VED of 3.42 J/mm³ with a low laser power of 3.8W and low scanning speed of 44.5 mm/s to process PA12 and MWCNT-PA12 powders [21]. Since the other UTS values of PA12 parts were below 50 MPa using a powder layer thickness of 100 μm and a VED of below 1 J/mm^3^ during processing (Table 2), the authors [21] obtained a double UTS value compared to others that dominated the PCA results.

A factor 3 higher AED value results in a factor 2 increase of the UTS (Figure 14b). UTS dependence with AED might be due to the employment of higher values, leading to an increased melting of the particles or more pronounced powder sintering. This fact can lead to a lower part porosity and higher density. It should be considered that higher AED also may increase the surface roughness and decrease dimensional accuracy of as-built polymer parts, limiting a further increase in UTS.

Furthermore, if the powder size is 3 times larger, the UTS is reduced to half its value, Figure 14c. As seen in Table 2, evaluating the different polymer classes of TPU [35] and PA12 [7,21,27,70] can affect PCA correlations. TPU is a standard amorphous polymer with a UTS lower than 20MPa, and PA12 is a semi-crystalline engineering polymer with a UTS of higher than 40 MPa (Figure 12a).

Finally, a factor 2 increase of the powder size would require twice as much laser power for L-PBF processing, Figure 14d. An increase in powder size can result in thicker powder layer thickness, which requires higher laser powers to increase heat penetration depth.

It should be noted that these results are determined from the reported properties of eight different powder compositions. Further studies reporting the same material, process, and as-built part properties that provide a complete set of values are necessary to extend the PCA analysis results. By doing so, thousands of observations, i.e., polymer type or compositions, could be computed to improve the correlations between variables with statistical relevance. To achieve this, it is fundamental, as shown in this review, to standardize the minimal data set of material, processing, and as-built part parameters that are being reported. In conclusion, a PCA is a powerful tool to analyze the dependencies between properties to find an optimization route for the complex scenario of cross-correlated material, process, and built parts properties. The slopes in the PCA scatter plots can be regarded as effectivity factors which tell how strong a value change in one paremeter affects the other. As a result of evaluating eight powder feedstocks, it is shown that an increase in the ultimate tensile strength can be achieved by increasing powder layer thickness (effectivity factor 1.5) and AED (effectivity factor 0.75) or by decreasing the size of the powder feedstocks (effectivity factor 1.5). Additionally, a decrease in the powder feedstock particle size proportionally reduces the laser power required for the processing (effectivity factor 1).

## 7. Conclusions

Indexed scientific publications on L-PBF of polymer powder feedstocks have increased to over 600 in the last decade. Sixteen percent of those publications are evaluated in this study to extract the reported material, process, and as-built part properties for 257 polymer powder variants. The analysis reveals that within the reported polymer powder feedstock compositions, 60% are the semi-crystalline engineering polymers of PA12, PBT, PA11, PA6, and PHBV. A wide range of polymer types are studied. Lab-scale production lets researchers develop different polymer types of powder feedstocks for L-PBF, intending to transfer them to the industry. However, this development progress on new powders is still not fully transferable to industrial applications. Hence, the main influential role in increasing the variability of new polymer types and their composites for testing in L-PBF comes from research activities. In that sense, self-build machines have a share of 26% in our evaluation and are mainly used for research purposes like testing new powder feedstocks, process parameters, and building functional parts. As the most-reported feedstock material, PA12 (including modified variants with particulate additives) has a share of 50% within all reported polymer compositions. The next most frequently reported polymer powder feedstocks is PEEK, with a share of 12% and the highest mechanical performance, and TPU, with a share of 6% and excellent elastomeric behavior of the 3D printed parts.

The polymer powder feedstocks’ material properties are also evaluated, finding that the powder size, flowability, and thermal behavior of polymer powder feedstocks are the most reported powder properties. The mean, D10, D50, and D90 are mostly reported for powder sizes with average values of 45 µm, 33 µm, 65 µm, and 108 µm, respectively. The Hausner ratio and angle of repose are the parameters most often employed to characterize the flowability, while the sintering window characterizes the material’s thermal response. The Hausner ratio for the evaluated polymers ranges from 1.08 to 1.33, with an average value of 1.23, marking the transition from poor to good powder flowability. The angle of repose also supports this trend, finding a variation between 33° to 53° with an average value of 45°. The sintering window depends on the molecular weight, crystallinity degree, and aging degree of the employed polymer. Consequently, the values reported for the sintering window ranged from 11 °C for PA11 to 37 °C for PA6.

The increasingly frequent use of additives to modify the properties of feedstock powders has been analyzed. The modification of the polymer powders by carbon-based additives is the most common strategy in L-PBF to enhance mechanical performance. Adding small amounts of CNT or MWCNT (<1 wt.%) to PA12 powder feedstocks increases the average UTS of as-built parts by a factor of 2, making PA12 additivated polymers compete with PEEK parts in terms of the UTS. The statistical analysis reveals that additives are used with a weight loading ranging from 0.1 to 80 wt.% in polymer powder feedstocks. Mostly C-based additives are employed compared to other material classes of additives, and CF, Ca-phosphate, and SiO_2_ are the alternative additives most used in the studies.

Different additives modify the material properties and built parts, depending on the application and the polymer class required. For example, CF is used to enhance the mechanical properties of semi-crystalline engineering and high-temperature polymers; Ca-phosphate is used in PCL, PHBV, and PDLLA to improve the biocompatibility of these biodegradable polymers; SiO_2_ is used to enhance the flowability and mechanical properties of several polymer powder feedstocks. In that sense, future developments on nano-additivation of the polymer powder feedstocks have a high potential to develop new powder feedstocks for L-PBF of polymers and built parts with enhanced performance and new functionalities.

As expected, laser power, scanning speed, hatch spacing, and powder layer thickness are the most reported variables of process properties in the literature. However, a general optimization route for each polymer powder material is still required for L-PBF. As a general approach, the volumetric and areal energy densities are calculated as a reference to achieve repeatability of the process for different L-PBF systems. The distribution of values reported for the processing parameters is still wide except for the powder layer thickness, with an average of 100 µm. Reducing powder layer thickness well below 150 µm is a trend to keep good processability for the reported powder sizes. The average values are as follows: laser power, 18 W; scanning speed, 2330 mm/s; powder layer thickness, 106 µm; hatch spacing, 200 µm; calculated volumetric energy density, 0.66 J/mm^3^; and calculated areal energy density, 0.075 J/mm^2^, as obtained from the evaluation of the top 100 cited references. For the most used polymer PA12, these values are 20 W, 3000 mm/s, 112 µm, 200 µm, 0.75 J/mm^3^, and 0.07 J/mm^2^, respectively.

The disparity of material properties and process parameters resulted in a wide variety of as-built part properties. The most-reported as-built part parameters were the tensile test, flexural test, and volumetric porosity. A wide range of volumetric porosity contents is found in the parts for different polymer types, except S-H polymers. Since an increase in porosity content decreases the mechanical properties of the sintered parts, further studies should focus on optimizing material properties and energy densities to reduce porosity contents in the as-built parts. The average UTS, elastic modulus, and elongation for the reported polymer parts were 42 MPa, 2.6 GPa, and 57%, respectively. For PA12, these values were 48 MPa, 2.7 GPa, and 17%. As-built polymer parts and PA12 parts have the same average flexural strength of 78 MPa and flexural modulus of 2.4 GPa.

The incorporation of additives is observed to enhance the as-built part properties significantly. Specifically, carbon-based additives were found to increase tensile and flexural strength. The highest UTS of 110 MPa was determined for 10 wt.% CF-PEEK parts, and the highest flexural strength of 183 MPa was determined for the 5 wt.% CF-PEEK parts. Fiber or tubular C-based additives resulted in the highest tensile and flexural strengths. This effect and the dependence with the volume fraction added to the polymer matrix should be further investigated for other types of fiber additives. The additivated PA12 polymers can reach the UTS of S-H polymers, opening up the possibility to employ PA12 parts in applications demanding under tensile stresses. However, S-H polymer parts are still the most resistant material under flexural stresses. The lowest mechanical strengths and elastic modulus with the highest ductility are obtained for amorphous standard polymers. In contrast, the semi-crystalline high-performance polymers exhibit the highest mechanical strength and elastic modulus but the lowest ductility. These S-H polymers also resulted in the highest densifications of over 99%.

Finally, a computational exploratory data analysis using PCA has been carried out to determine the correlation strength between reported properties. A big data matrix composed of 257 powder compositions with their corresponding reported properties is evaluated. Due to the data matrix’s mismatching property values, the correlation matrix between powder size, laser power, scanning speed, hatch spacing, powder layer thickness, UTS, and elongation is obtained for only eight powder compositions, where two of them were TPU, and the rest was PA12. TPU exhibits a UTS lower than 20 MPa with large elongation 300%, while for PA12, the UTS is over 40 MPa and the elongation is below 20%. These vast differences might dominate the PCA results. The correlation obtained shows that increasing the powder layer thickness as well as the AED, and decreasing the powder size leads to an increased UTS, with effectivity factors reaching 1.5. It is clear that PCA is useful to evaluate the relations between investigated variables, but for an excellent statistical relevance of the PCA results, the number of publications that report the complete process and material datasets must be increased, as well as researchers’ awareness of the importance of reporting full parameter sets.

## Figures and Tables

**Table 1:** Correlation matrix of PCA for L-PBF of polymer powder feedstocks.

	Powder Size	VED	AED	Laser Power	Scanning Speed	Layer Thickness	Hatch Spacing	UTS	Elongation
**Powder Size**	1	0.09922	−0.03446	0.39481	−0.23362	−0.14307	0.02324	−0.54097	0.28128
**VED**	0.09922	1	0.98235	−0.72197	−0.84689	0.94208	−0.49303	0.67412	−0.26240
**AED**	−0.03446	0.98235	1	−0.81100	−0.78176	0.98819	−0.38709	0.78199	−0.30151
**Laser Power**	0.39481	−0.72197	−0.81100	1	0.63875	−0.86263	0.22207	−0.74786	−0.02901
**Scanning Speed**	−0.23362	−0.84689	−0.78176	0.63875	1	−0.70787	0.71575	−0.31273	−0.17942
**Layer Thickness**	−0.14307	0.94208	0.98819	−0.86263	−0.70787	1	−0.29013	0.84981	−0.32563
**Hatch Spacing**	0.02324	−0.49303	−0.38709	0.22207	0.71575	−0.29013	1	−0.10027	−0.00206
**UTS**	−0.54097	0.67412	0.78199	−0.74786	−0.31273	0.84981	−0.10027	1	−0.61968
**Elongation**	0.28128	−0.26240	−0.30151	−0.02901	−0.17942	−0.32563	−0.00206	−0.61968	1

**Table 2:** Data matrix of the PCA.

Ref.	Polymer Type	Additives	D50 (μm)	VED (J/mm³)	AED (J/mm²)	Laser Power (W)	Scanning Speed (mm/s)	Layer Thickness (µm)	Hatch Spacing (µm)	UTS (MPa)	Elongation (%)
[71]	aged PA12		53	0.15	0.02	18	4000	100	300	48	47
[70]	PA12		56	0.36	0.04	18	2500	100	200	50	18
[35]	TPU		63	0.67	0.07	10	1000	100	150	18	559
[27]	PA12		37	0.90	0.09	18	2000	100	100	38	21
[27]	PA12	silica	37	1.00	0.1	20	2000	100	100	46	20
[35]	TPU		200	1.60	0.16	24	1000	100	150	4	208
[21]	PA12		58	3.41	0.68	4	45	200	125	86	11
[21]	PA12	MWCNT	58	3.41	0.68	4	45	200	125	94	9

## Data Availability

The data presented in this study are available on request from the corresponding author. The data are not publicly available due to project restrictions.

## Supplementary Materials

The following are available online at https://www.mdpi.com/1996-1944/14/5/1169/s1, Table S1: Web of Science search results for L-PBF of several polymer types between 2009 and 2019.
