# Supplementary material for: Laser Powder Bed Fusion of Polymers: Quantitative Research Direction Indices
Source: Materials (Basel). 2021 Mar 2;14(5):1169. doi: 10.3390/ma14051169 (PMC7958861; doi:10.3390/ma14051169)
Supplement: Supplementary file 1 [file materials-14-01169-s001.pdf]

materials

MDPI

Supplementary Information

Laser Powder Bed Fusion of Polymers: Quantitative Research Direction Indices

Ihsan Murat Kusoglu [1], Carlos Doñate-Buendía [1,2], Stephan Barcikowski [1,*] and Bilal Gökce [1,2]

[1] Technical Chemistry I, Center for Nanointegration Duisburg-Essen (CENIDE), University of Duisburg, 45141 Essen, Germany; ihsan.kusoglu@uni-due.de (I.M.K.), carlos.donate-buendia@uni-due.de (C.D.-B.), bilal.goekce@uni-due.de (B.G.)
[2] Materials Science and Additive Manufacturing, School of Mechanical Engineering and Safety Engineering, University of Wuppertal, 42119 Wuppertal, Germany
* Correspondence: stephan.barcikowski@uni-due.

Citation: Kusoglu, I.M.; Doñate-Buendía, C.; Barcikowski, S.; Gökce, B. Laser Powder Bed Fusion of Polymers: Quantitative Research Direction Indices. *Materials*, **2021**, 14, 1169. https://doi.org/10.3390/ma14051169

Academic Editor: Aniello Riccio

Received: 21 January 2021
Accepted: 24 February 2021
Published: 2 March 2021

Publisher's Note: MDPI stays neutral with regard to jurisdictional claims in published maps and institutional

Copyright: © 2021 by the authors. Submitted for possible open access publication under the terms and conditions of the Creative Commons Attribution (CC BY) license (http://creativecommons.org/licenses/by/4.0/).

Table S1. Web of Science search results for L-PBF of several polymer types between 2009 and 2019.

Search Set	Search String TOPIC:	Results Found	Sum of the Times Cited	Average Citations per Item	h-index
#1	additive manufacture* AND polymer	2429	42496	17.5	84
#2	powder AND bed AND fuse* AND polymer	48	827	17.23	10
#3	selective AND laser AND sinter* (NOT melt*)	280	4867	17.38	37
#4	selective AND laser AND melt* AND polymer (NOT sinter*)	82	1664	20,29	22
#5	laser AND sinter* AND polymer (NOT melt* OR selective)	173	2448	14.15	25
#6	laser AND beam AND melt* AND polymer (NOT sinter* OR selective OR join* OR weld* OR ablation)	46	530	11.52	13
#7	#2 OR #3 OR #4 OR #5 OR #6	612	10116	16.53	46
#8	#2 AND (polyamide 12 OR PA12)	13	81	6.23	6
#9	#3 AND (polyamide 12 OR PA12	106	1032	9.74	18

#10	#4 AND (polyamide 12 OR PA12	2	10	5	2
#11	#5 AND (polyamide 12 OR PA12)	41	321	7.83	10
#12	#6 AND (polyamide 12 OR PA12)	5	33	6.6	4
#13	#8 OR #9 OR #10 OR #11 OR #12	160	1414	8.84	19
#14	#2 AND (Polyether AND ether AND ketone OR PEEK)	1	2	2	1
#15	#3 AND (Polyether AND ether AND ketone OR PEEK)	19	173	9.11	6
#16	#4 AND (Polyether AND ether AND ketone OR PEEK)	9	40	4.44	3
#17	#5 AND (Polyether AND ether AND ketone OR PEEK)	13	227	17.46	10
#18	#6 AND (Polyether AND ether AND ketone OR PEEK)	1	14	14	1
#19	#14 OR #15 OR #16 OR#17 OR #18	43	456	10.6	14
#20	#2 AND (polypropylene OR PP)	3	80	26.67	3
#21	#3 AND (polypropylene OR PP)	26	375	14.42	9
#22	#4 AND (polypropylene OR PP)	21	522	24.86	10
#23	#5 AND (polypropylene OR PP)	14	203	14.5	7
#24	#6 AND (polypropylene OR PP)	13	131	10.08	6
#25	#20 OR #21 OR #22 OR #23 OR #24	75	1296	17.28	20
#26	#2 AND (Polyetherketone OR PEK)	1	5	5	1
#27	#3 AND (Polyetherketone OR PEK)	6	61	10.17	4
#28	#4 AND (Polyetherketone OR PEK)	0	0	0	0
#29	#5 AND (Polyetherketone OR PEK)	8	143	17.88	6
#30	#6 AND (Polyetherketone OR PEK)	0	0	0	0
#31	#26 OR #27 OR #28 OR #29 OR #30	15	209	13.93	9
#32	#2 AND (Polyethylene OR PE OR HDPE)	6	144	24	4
#33	#3 AND (Polyethylene OR PE OR HDPE)	26	687	26.42	12
#34	#4 AND (Polyethylene OR PE OR HDPE)	10	51	5.1	4
#35	#5 AND (Polyethylene OR PE OR HDPE)	28	435	15.54	11
#36	#6 AND (Polyethylene OR PE OR HDPE)	9	105	11.67	5
#37	#32 OR #33 OR #34 OR #35 OR #36	75	1309	17	19
#38	#2 AND (polyamid* 6 OR PA6)	0	0	0	0
#39	#3 AND (polyamid* 6 OR PA6)	9	88	9.78	4
#40	#4 AND (polyamid* 6 OR PA6)	0	0	0	0
#41	#5 AND (polyamid* 6 OR PA6)	3	27	9	2
#42	#6 AND (polyamid* 6 OR PA6)	1	2	2	1
#43	#38 OR #39 OR #40 OR #41 OR #42	13	117	9	4
#44	#2 AND (polyamid* 11 OR PA11)	0	0	0	0
#45	#3 AND (polyamid* 11 OR PA11)	6	63	10.5	4
#46	#4 AND (polyamid* 11 OR PA11)	0	0	0	0
#47	#5 AND (polyamid* 11 OR PA11)	1	9	9	1
#48	#6 AND (polyamid* 11 OR PA11)	0	0	0	0
#49	#44 OR #45 OR #46 OR #47 OR #48	7	72	10.29	5
#50	#2 AND (polybutylene terephthalate OR PBT)	1	4	4	1
#51	#3 AND (polybutylene terephthalate OR PBT)	4	49	12.25	4
#52	#4 AND (polybutylene terephthalate OR PBT)	1	30	30	1
#53	#5 AND (polybutylene terephthalate OR PBT)	0	0	0	0
#54	#6 AND (polybutylene terephthalate OR PBT)	1	8	8	1
#55	#50 OR #51 OR #52 OR #53 OR #54	7	97	13.9	7
#56	#2 AND (Thermoplastic polyurethane OR TPU)	1	1	1	1
#57	#3 AND (Thermoplastic polyurethane OR TPU)	8	151	18.9	7
#58	#4 AND (Thermoplastic polyurethane OR TPU)	1	1	1	1
#59	#5 AND (Thermoplastic polyurethane OR TPU)	4	38	9.5	3
#60	#6 AND (Thermoplastic polyurethan OR TPU)	2	28	14	2
#61	#56 OR #57 OR #58 OR #59 OR #60	15	218	14.5	8
#62	#2 AND (Polystyrene OR PS)	5	19	3.8	2
#63	#3 AND (Polystyrene OR PS)	36	430	11.9	10
#64	#4 AND (Polystyrene OR PS)	18	253	14	8
#65	#5 AND (Polystyrene OR PS)	28	386	13.8	11

#66	#6 AND (Polystyrene OR PS)	11	128	11.6	7
#67	#62 OR #63 OR #64 OR #65 OR #66	96	1201	12.5	17
